# Supplementary material for: The Effect of Carbohydrate Restriction on Lipids, Lipoproteins, and Nuclear Magnetic Resonance-Based Metabolites: CALIBER, a Randomised Parallel Trial
Source: Nutrients. 2023 Jun 30;15(13):3002. doi: 10.3390/nu15133002 (PMC10347188; doi:10.3390/nu15133002)
Supplement: Supplementary file 1 [file nutrients-15-03002-s001.zip › nutrients-2437029-supplementary.pdf]

Table S1. The effect of a LCHF and HCLF diet on markers of cardiovascular disease.

	LCHF				HCLF				*p* value		
	**Week 0**	**Week 4**	**Week 8**	**95% CI difference 0-8 weeks**	**Week 0**	**Week 4**	**Week 8**	**95% CI Difference 0-8 weeks**	**Group**	**Time**	**Time × Diet**
**VLDL lipids**											
Total lipids in VLDL (mmol/L)	2.11 ± 0.95	1.93 ± 0.90	2.06 ± 0.77	-0.44, 0.35	1.96 ± 0.65	2.05 ± 0.59	1.94 ± 0.73	-0.28, 0.25	0.982	0.542	0.221
Total lipids in chylomicrons and extremely large VLDL (mmol/L)	0.21 ± 0.22	0.11 ± 0.11	0.17 ± 0.17	-0.14, 0.05	0.19 ± 0.11	0.15 ± 0.11	0.14 ± 0.13	-0.10, 0.00	0.945	0.064	0.384
Total lipids in very large VLDL (mmol/L)	0.19 ± 0.14	0.15 ± 0.11	0.16 ± 0.10	-0.10, 0.03	0.18 ± 0.09	0.18 ± 0.08	0.17 ± 0.10	-0.04, 0.02	0.813	0.186	0.065
Total lipids in large VLDL (mmol/L)	0.31 ± 0.17	0.27 ± 0.17	0.27 ± 0.15	-0.13, 0.05	0.30 ± 0.012	0.32 ± 0.12	0.31 ± 0.15	-0.04, 0.06	0.690	0.424	0.055
Total lipids in medium VLDL (mmol/L)	0.61 (0.34)	0.60 (0.52)	0.61 (0.29)	-0.10, 0.12	0.60 (0.17)	0.67 (0.08)	0.63 (0.25)	-0.05, 0.12	0.339	0.578	0.562
Total lipids in small VLDL (mmol/L)	0.43 (0.21)	0.42 (0.30)	0.43 (0.19)	-0.06, 0.07	0.44 (0.10)	0.46 (0.09)	0.41 (0.16)	-0.04, 0.05	0.362	0.409	**0.034**
Total lipids in very small VLDL (mmol/L)	0.39 (0.13)	0.43 (0.18)	0.44 (0.11)	0.01. 0.09	0.35 (0.05)	0.36 (0.06)	0.36 (0.10)	-0.05, 0.04	0.687	0.725	**0.045**
**IDL lipids**											
Total lipids in IDL (mmol/L)	1.38 ± 0.26	1.51 ± 0.32 [a]	1.55 ± 0.20 [a]	0.05, 0.28	1.18 ± 0.28	1.31 ± 0.24	1.27 ± 0.31	-0.02, 0.21	0.175	**0.012**	0.548
**LDL lipids**											
Total lipids in LDL (mmol/L)	2.96 ± 0.60	3.16 ± 0.70	3.12 ± 0.50	-0.06, 0.38	2.53 ± 0.67	2.87 ± 0.61 [a]	2.81 ± 0.75 [a]	0.09, 0.46	0.375	**0.010**	0.663
Total lipids in large LDL (mmol/L)	1.87 ± 0.37	2.03 ± 0.43	2.01 ± 0.30	-0.01, 0.28	1.60 ± 0.43	1.82 ± 0.39 [a]	1.78 ± 0.48 [aa]	0.06, 0.31	0.337	**0.005**	0.861
Total lipids in medium LDL (mmol/L)	0.72 (0.28)	0.80 (0.33)	0.72 (0.23)	-0.05, 0.08	0.65 (0.14)	0.77 (0.14)	0.74 (0.18)	0.02, 0.11	0.944	0.214	0.972
Total lipids in small LDL (mmol/L)	0.32 (0.11)	0.36 (0.12)	0.32 (0.07)	-0.02, 0.04	0.29 (0.05)	0.33 (0.05)	0.33 (0.07)	0.01, 0.04	0.936	0.302	0.694
**HDL lipids**											
Total lipids in HDL (mmol/L)	2.70 (0.40)	2.47 (0.99)	2.65 (0.43)	0.00, 0.00	2.79 (0.97)	2.76 (0.63)	2.97 (1.03)	0.00, 0.00	0.795	0.324	0.809
Total lipids in very large HDL (mmol/L)	0.14 ± 0.06	0.18 ± 008	0.19 ± 0.11	-0.03, 0.11	0.14 ± 0.08	0.14 ± 0.08	0.15 ± 0.09	-0.03, 0.05	0.601	0.156	**0.021**
Total lipids in large HDL (mmol/L)	0.41 (0.44)	0.44 (0.59)	0.41 (0.37)	-0.13, 0.39	0.48 (0.38)	0.43 (0.49)	0.50 (0.58) [bb]	-0.08, 0.24	0.881	0.123	0.065
Total lipids in medium HDL (mmol/L)	0.90 (0.11)	0.82 (0.31)	0.91 (0.13)	-0.14, 0.17	0.98 (0.27)	0.94 (0.16)	1.03 (0.33)	-0.04, 0.20	0.521	0.424	0.915
Total lipids in small HDL (mmol/L)	1.16 ± 0.11	1.11 ± 0.11	1.13 ± 0.08	-0.11, 0.03	1.14 ± 0.14	1.19 ± 0.16	1.20 ± 0.18	0.00, 0.12	0.417	0.881	0.078

**Particles**											
Total concentration of lipoprotein particles (µmol/L)	17.01 ± 1.34	17.22 ± 2.23	17.37 ± 1.97	-0.86, 1.57	16.50 ± 2.27	17.49 ± 2.60	17.82 ± 3.51	0.11, 2.54	0.807	0.363	0.827
Apolipoprotein B (g/L)	0.94 (0.31)	1.10 (0.42)	0.99 (0.27)	-0.01, 0.16	0.87 (0.17)	0.98 (0.14) [aa]	0.95 (0.20) [ab]	-0.03, 0.12	0.426	0.052	**<0.001**
Apolipoprotein A1 (g/L)	1.35 (0.12)	1.29 (0.34)	1.36 (0.17)	-0.13, 0.20	1.41 (0.34)	1.38 (0.18)	1.46 (0.37)	-0.03, 0.22	0.697	0.410	0.993
Ratio of apolipoprotein B to apolipoprotein A1	0.71 ± 0.19	0.78 ± 0.28	0.77 ± 0.22	-0.05, 0.16	0.63 ± 0.16	0.66 ± 0.13	0.63 ± 0.17	-0.08, 0.08	0.277	**0.043**	0.912
**VLDL particles**											
Concentration of VLDL particles (µmol/L)	0.15 (0.06)	0.16 (0.01)	0.15 (0.07)	-0.12, 0.08	0.15 (0.03)	0.16 (0.03)	0.15 (0.05)	-0.02, 0.02	0.602	0.876	0.808
Average diameter for VLDL particles (nm)	38.79 (1.59)	37.84 (2.03)	38.22 (1.79)	-1.42. 0.47	38.82 (0.65)	38.98 (1.07)	39.15 (1.42)	-0.36, 0.28	0.624	0.543	0.484
Concentration of chylomicrons and extremely large VLDL particles (nmol/L)	1.06 (2.43)	0.56 (1.59)	1.19 (1.53)	-1.06, 0.39	1.50 (0.71)	1.07 (1.16)	0.89 (1.71)	-0.71, 0.01	0.862	0.077	0.280
Concentration of very large VLDL particles (nmol/L)	3.36 ± 2.40	2.56 ± 1.96	2.84 ± 1.83	-0.54, 1.59	3.11 ± 1.52	3.16 ± 1.37	2.96 ± 1.80	-0.36, 0.66	0.818	0.190	0.055
Concentration of large VLDL particles (nmol/L)	9.96 ± 5.86	8.28 ± 5.32	8.73 ± 4.70	-3.96, 1.50	9.50 ± 3.88	10.03 ± 3.68	9.58 ± 4.74	-1.38, 1.55	0.711	0.374	**0.050**
Concentration of medium VLDL particles (nmol/L)	38.72 (22.13)	40.15 (31.06)	40.07 (19.88)	-3.52, 9.69	36.44 (11.84)	41.72 (4.63)	38.07 (13.21)	-3.59, 6.96	0.592	0.787	0.679
Concentration of small VLDL particles (nmol/L)	40.33 ± 15.00	39.03 ± 15.86	40.82 ± 11.99	-5.95, 6.92	39.07 ± 11.64	40.89 ± 9.75	39.34 ± 12.67	-4.57, 5.11	0.933	0.611	0.459
Concentration of very small VLDL particles (nmol/L)	56.81 ± 13.66	60.01 ± 16.77	64.99 ± 11.08 [a]	2.30, 14.07	51.81 ± 11.61	54.16 ± 9.83	51.10 ± 11.07	-6.66, 5.23	0.297	0.319	0.064
**IDL particles**											
Concentration of IDL particles (nmol/L)	355.66 ± 80.32	375.13 ± 98.75	398.13 ± 71.49	5.75, 79.19	304.88 ± 71.55	341.87 ± 55.61	320.06 ± 69.45	-22.80, 53.16	0.237	0.056	0.321
**LDL particles**											
Concentration of LDL particles (µmol/L)	1.36 (0.45)	1.54 (0.56)	1.39 (0.35)	-0.03, 0.22	1.26 (0.25)	1.40 (0.20)	1.36 (0.24)	-0.01, 0.17	0.706	0.547	0.895
Average diameter for LDL particles (nm)	23.95 ± 0.08	23.99 ± 0.07	23.97 ± 0.06	-0.03, 0.07	23.93 ± 0.10	23.96 ± 0.04	23.93 ± 0.07	-0.06, 0.07	0.374	0.080	0.980
Concentration of large LDL particles (nmol/L)	856.47 (248.22)	992.52 (318.87)	864.58 (183.96)	-24.73, 155.73	796.64 (167.44)	871.60 (114.85)	838.08 (161.00)	-17.37, 120.74	0.576	0.470	0.992
Concentration of medium LDL particles (nmol/L)	325.11 (136.82)	348.89 (169.21)	333.86 (122.41)	-16.27, 47.74	315.94 (70.73)	340.78 (53.13)	348.07 (74.69)	-9.62, 36.20	0.665	0.428	0.801
Concentration of small LDL particles (nmol/L)	181.30 (63.31)	198.88 (70.46)	187.76 (43.50)	-4.56, 25.29	166.06 (31.30)	190.80 (26.94)	190.15 (56.30)	1.82, 24.24	0.693	0.791	0.964
**HDL particles**											

Concentration of HDL particles (µmol/L)	15.16 (1.44)	14.80 (2.54)	15.14 (1.40)	-1.06, 1.48	14.39 (3.06)	15.75 (2.84)	15.53 (4.12)	0.03, 2.44	0.768 [Υ]	0.964 [Υ]	ns
Average diameter for HDL particles (nm)	9.55 ± 0.20	9.59 ± 0.24	9.62 ± 0.27	-0.07, 0.21	9.58 ± 0.24	9.56 ± 0.23	9.60 ± 0.24	-0.07, 0.11	0.955	0.162	0.047
Concentration of very large HDL particles (µmol/L)	0.21 ± 0.07	0.26 ± 0.09	0.26 ± 0.12	-0.03, 0.13	0.21 ± 0.09	0.21 ± 0.10	0.22 ± 0.10	-0.03, 0.060	0.566	0.154	**0.031**
Concentration of large HDL particles (µmol/L)	0.89 (1.01)	0.95 (1.30)	0.95 (0.80)	-0.27, 0.92	1.07 (0.96)	0.95 (1.22)	1.09 (1.37) [bb]	-0.16, 0.57	0.918	0.105	**0.043**
Concentration of medium HDL particles (µmol/L)	3.40 (0.40)	3.06 (1.34)	3.39 (0.56)	-0.51, 0.72	3.69 (1.27)	3.57 (0.67)	3.93 (1.36)	-0.13, 0.83	0.521	0.424	0.915
Concentration of small HDL particles (µmol/L)	10.20 ± 0.98	10.00 ± 0.85	9.93 ± 0.74	-0.81, 0.28	9.81 ± 1.33	10.47 ± 1.49 [a]	10.48 ± 1.76 [a]	0.12, 1.21	0.621	0.672	0.067
**Fatty acids, amino acids, and metabolic biomarkers**											
Phosphoglycerides (mmol/L)	2.25 ± 0.26	2.26 ± 0.31	2.34 ± 0.29	-0.12, 0.30	2.15 ± 0.32	2.26 ± 0.39	2.31 ± 0.50	-0.05, 0.38	0.994	0.435	0.991
Ratio of triglycerides to phosphoglycerides	0.52 ± 0.22	0.44 ± 0.18	0.46 ± 0.15	-0.17, 0.05	0.54 ± 0.18	0.51 ± 0.13	0.50 ± 0.20	-0.08, 0.01	0.607	0.088	0.169
Total cholines (mmol/L)	2.56 ± 0.25	2.61 ± 0.32	2.68 ± 0.29	-0.09, 0.32	2.45 ± 0.34	2.59 ± 0.41	2.63 ± 0.53	-0.04, 0.41	0.931	0.307	0.997
Phosphatidylcholines (mmol/L)	2.05 ± 0.22	2.07 ± 0.32	2.13 ± 0.28	-0.10, 0.26	1.94 ± 0.31	2.08 ± 0.39	2.13 ± 0.50	-0.03, 0.42	0.942	0.338	0.906
Sphingomyelins (mmol/L)	0.48 ± 0.04	0.52 ± 0.04 [aa]	0.52 ± 0.05	0.00, 0.08	0.44 ± 0.07	0.47 ± 0.06	0.47 ± 0.09	0.00, 0.06	0.240	**0.007**	0.285
Total fatty acids (mmol/L)	12.08 ± 2.57	11.78 ± 1.70	12.15 ± 1.36	-1.19, 1.34	11.4 ± 2.06	11.7 ± 1.93	11.82 ± 2.39	-0.51, 1.33	0.876	0.871	0.686
Degree of unsaturation	1.35 (0.05)	1.39 (0.06)	1.38 (0.02)	0.00, 0.01	1.38 (0.05)	1.35 (0.03)	1.35 (0.05)	-0.04, 0.06	0.711	0.578	0.762
Omega-3 fatty acids (mmol/L)	0.36 (0.11)	0.47 (0.07)	0.42 (0.10)	-0.08, 0.20	0.38 (0.13)	0.44 (0.20)	0.53 (0.32)	-0.03, 0.15	0.915	0.178	0.260
Omega-6 fatty acids (mmol/L)	4.76 ± 0.67	4.73 ± 0.60	4.9 ± 0.47	-0.17, 0.45	4.51 ± 0.67	4.69 ± 0.69	4.76 ± 0.81	-0.19, 0.68	0.810	0.546	0.769
Polyunsaturated fatty acids (mmol/L)	5.15 ± 0.74	5.2 ± 0.61	5.34 ± 0.48	-0.16, 0.55	4.88 ± 0.73	5.09 ± 0.76	5.19 ± 0.97	-0.21, 0.82	0.776	0.421	0.886
Monounsaturated fatty acids (mmol/L)	2.91 ± 0.97	2.62 ± 0.53	2.75 ± 0.43	-0.65, 0.34	2.81 ± 0.78	2.79 ± 0.60	2.82 ± 0.74	-0.23, 0.25	0.763	0.217	0.399
Saturated fatty acids (mmol/L)	4.03 ± 0.91	3.96 ± 0.61	4.06 ± 0.51	-0.50, 0.57	3.71 ± 0.72	3.83 ± 0.62	3.8 ± 0.77	-0.17, 0.36	0.642	0.866	0.861
Linoleic acid (mmol/L)	3.81 ± 0.60	3.66 ± 0.59	3.81 ± 0.48	-0.30, 0.32	3.49 ± 0.63	3.72 ± 0.72	3.84 ± 0.84	-0.13, 0.81	0.988	0.488	0.300
Docosahexaenoic acid (mmol/L)	0.18 ± 0.03	0.25 ± 0.02 [aaa]	0.23 ± 0.06	-0.01, 0.11	0.19 ± 0.04	0.21 ± 0.05	0.22 ± 0.07	-0.01, 0.07	0.634	**0.013**	0.206
Ratio of omega-3 fatty acids to total fatty acids (%)	3.19 ± 0.51	4.06 ± 0.89	3.62 ± 0.90	-0.56, 1.42	3.25 ± 0.58	3.42 ± 0.95	3.53 ± 0.96	-0.35, 0.90	0.462	0.131	0.308
Ratio of omega-6 fatty acids to total fatty acids (%)	40.01 ± 3.35	40.29 ± 1.24	40.44 ± 1.60	-2.49, 3.35	39.95 ± 3.84	40.2 ± 1.46	40.59 ± 2.36	-0.81, 2.08	0.917	0.410	0.990
Ratio of polyunsaturated fatty acids to total fatty acids (%)	43.20 ± 3.50	44.35 ± 2.00	44.05 ± 1.21	-1.64, 3.35	43.21 ± 3.77	43.62 ± 1.55	44.11 ± 1.85	-0.86, 2.68	0.752	0.150	0.745
Ratio of monounsaturated fatty acids to total fatty acids (%)	23.55 ± 2.99	22.07 ± 1.56	22.56 ± 1.28	-2.65, 0.66	24.28 ± 3.51	23.66 ± 2.01	23.63 ± 2.63	-1.41, 0.11	0.310	**0.026**	0.340

Ratio of saturated fatty acids to total fatty acids (%)	33.00 (1.33)	32.96 (1.40)	33.21 (2.09)	-1.15, 1.42	32.28 (2.09)	32.62 (0.77) [a]	32.77 (1.64)	-1.45, 0.93	0.692	**0.044**	0.101
Ratio of linoleic acid to total fatty acids (%)	31.88 ± 2.61	31.03 ± 1.78	31.42 ± 2.40	-3.24, 2.31	30.78 ± 2.99	31.65 ± 1.78	32.43 ± 2.01	-0.10, 3.40	0.849	0.366	0.392
Ratio of docosahexaenoic acid to total fatty acids (%)	1.44 (0.67)	1.94 (0.53) [aa]	1.71 (0.74)	-0.14, 0.80	1.62 (0.54)	1.86 (0.67)	1.91 (0.25)	-0.15, 0.44	0.822	**0.005**	0.080
Ratio of polyunsaturated fatty acids to monounsaturated fatty acids	1.88 ± 0.38	2.02 ± 0.23	1.96 ± 0.13	-0.13, 0.30	1.83 ± 0.41	1.86 ± 0.23	1.89 ± 0.28	-0.04, 0.17	0.446	0.086	0.340
Ratio of omega-6 fatty acids to omega-3 fatty acids	12.89 (2.42)	10.31 (2.15)	12.70 (3.78)	-4.56, 2.58	11.68 (2.79)	11.62 (4.22)	9.95 (6.47)	-2.27, 1.96	0.514	0.280	0.306
Alanine (mmol/L)	0.33 ± 0.06	0.29 ± 0.07	0.30 ± 0.05	-0.09, 0.04	0.37 ± 0.07	0.37 ± 0.12	0.38 ± 0.13	-0.07, 0.10	0.094	0.474	0.314
Glutamine (mmol/L)	0.60 ± 0.05	0.52 ± 0.07 [aa *]	0.58 ± 0.07	-0.09, 0.06	0.64 ± 0.06	0.62 ± 0.07	0.65 ± 0.06	-0.05, 0.07	**0.016**	**0.031**	0.203
Glycine (mmol/L)	0.17 ± 0.06	0.17 ± 0.05	0.17 ± 0.04	-0.04, 0.04	0.19 ± 0.05	0.19 ± 0.05	80.22 ± 0.09	-0.02, 0.08	0.239	0.137	0.730
Histidine (mmol/L)	0.07 ± 0.01	0.07 ± 0.01	0.07 ± 0.01 [aa]	-0.01, 0.00	0.07 ± 0.01	0.07 ± 0.01	0.08 ± 0.01	0.00, 0.02	0.190	0.735	0.070
Total concentration of branched-chain amino acids (leucine + isoleucine + valine) (mmol/L)	0.42 ± 0.08	0.45 ± 0.05 *	0.47 ± 0.08 *	-0.05, 0.15	0.39 ± 0.05	0.38 ± 0.05	0.38 ± 0.04	-0.02, 0.02	**0.022**	0.444	0.445
Isoleucine (mmol/L)	0.06 ± 0.02	0.06 ± 0.01	0.07 ± 0.02	-0.02, 0.04	0.06 ± 0.01	0.06 ± 0.01	0.05 ± 0.01	-0.01, 0.01	0.077	0.575	0.517
Leucine (mmol/L)	0.12 ± 0.02	0.13 ± 0.02	0.14 ± 0.03	-0.02, 0.05	0.11 ± 0.02	0.11 ± 0.02	0.11 ± 0.02	-0.01, 0.01	0.103	0.449	0.600
Valine (mmol/L)	0.23 ± 0.04	0.26 ± 0.04 *	0.26 ± 0.02 **	-0.02, 0.07	0.22 ± 0.03	0.21 ± 0.02	0.22 ± 0.02	-0.02, 0.02	**0.011**	0.422	0.278
Phenylalanine (mmol/L)	0.05 ± 0.01	0.05 ± 0.01	0.05 ± 0.01	-0.01, 0.00	0.05 ± 0.01	0.05 ± 0.01	0.05 ± 0.01	0.00, 0.01	0.263	0.334	0.088
Tyrosine (mmol/L)	0.07 ± 0.01	0.06 ± 0.02 [aa]	0.06 ± 0.01 [a]	-0.02, 0.00	0.07 ± 0.01	0.06 ± 0.01	0.06 ± 0.01	-0.01, 0.00	0.435	**0.001**	0.051
Glucose (mmol/L)	5.45 ± 0.42	5.37 ± 0.52	5.37 ± 0.56	-0.39, 0.23	5.5 ± 0.31	5.45 ± 0.64	5.50 ± 0.63	-0.43, 0.42	0.748	0.901	0.544
Lactate (mmol/L)	2.83 (3.73)	2.41 (2.47)	1.06 (1.27)	-2.81, 0.78	1.77 (4.01)	2.10 (3.34)	3.76 (4.47)	-2.51, 5.42	0.467	0.897	0.323
Pyruvate (mmol/L)	0.08 ± 0.04	0.09 ± 0.04	0.06 ± 0.03	-0.06, 0.02	0.09 ± 0.06	0.11 ± 0.07	0.11 ± 0.05	-0.04, 0.07	0.194	0.688	0.554
Citrate (mmol/L)	0.07 ± 0.02	0.08 ± 0.02 [aa *]	0.09 ± 0.01 [aaa **]	0.01, 0.03	0.07 ± 0.01	0.07 ± 0.01	0.07 ± 0.01	-0.02, 0.00	0.080	0.041	**<0.001**
3-Hydroxybutyrate (mmol/L)	0.07 (0.02)	0.32 (0.42) [aa] **	0.13 (0.18) [bb *]	-0.06, 0.24	0.08 (0.07)	0.07 (0.07)	0.06 (0.04)	-0.06, 0.03	**0.012**	**0.020**	**0.018**
Acetate (mmol/L)	0.04 (0.02)	0.05 (0.01)	0.06 (0.04)	-0.06, 0.16	0.06 (0.03)	0.05 (0.02)	0.05 (0.01)	-0.03, 0.01	0.516	0.827	0.177
Acetoacetate (mmol/L)	0.01 (0.01)	0.06 (0.08) [aa **]	0.03 (0.03) [bb]	-0.02, 0.04	0.01 (0.01)	0.01 (0.03)	0.02 (0.02)	-0.01, 0.02	**0.031**	0.193	**0.013**
Acetone (mmol/L)	0.03 (0.01)	0.08 (0.07)	0.07 (0.06) [aaa ***]	0.01, 0.11	0.02 (0.01)	0.03 (0.02)	0.02 (0.01)	-0.02, 0.00	**0.021**	**0.017**	**0.001**
Creatinine (μmol/L)	76.28 ± 10.62	73.03 ± 10.48 [a]	79.12 ± 11.35 *	-1.96, 7.65	80.12 ± 13.00	77.17 ± 12.81	78.09 ± 15.26	-7.18, 3.11	0.660	0.253	0.646
Albumin (g/L)	42.11 ± 2.68	43.44 ± 2.82 [a]	44.05 ± 1.93 [a]	0.15, 3.74	44.91 ± 1.66	45.49 ± 1.13	45.44 ± 2.05	-1.41, 2.47	0.057	**0.032**	0.152
Glycoprotein acetyls (mmol/L)	0.79 ± 0.11	0.75 ± 0.08	0.76 ± 0.08	-0.07, 0.02	0.75 ± 0.08	0.74 ± 0.07	0.75 ± 0.08	-0.05, 0.05	0.625	0.132	0.222

Values are expressed as means ± SD or median (interquartile range) of n=8 LCHF & n=8 HCLF. [a] $p < 0.05$, [aa] $P < 0.01$, [aaa] $p < 0.001$, denotes significant difference to week 0; [b] $p < 0.05$, [bb] $p < 0.01$, denotes significant difference to week 4, * $p < 0.05$, ** $p < 0.01$ denotes significant difference between groups at that timepoint. [ϒ] non-parametric (Friedman test) analysis for the effect of time. ns indicates there were no significant differences between groups at any time point by non-parametric test (Kruskal–Wallis test). Bold writing highlights significant results (p <0.0.5). CI, Confidence interval; HCLF, high carbohydrate low fat; LCHF, low carbohydrate high fat.
